# Comparison of Performance Score for Female and Male Residents in General Surgery Doing Supervised Real-Life Laparoscopic Appendectomy: Is There a Norse Shield-Maiden Effect?

**DOI:** 10.1007/s00268-020-05921-4

**Published:** 2021-01-18

**Authors:** Benedicte Skjold-Ødegaard, Hege Langli Ersdal, Jörg Assmus, Kjetil Soreide

**Affiliations:** 1 Faculty of Health Sciences, University of Stavanger, Stavanger, Norway; 2 Department of Gastrointestinal Surgery, Stavanger University Hospital, Stavanger, Norway; 3 Critical Care and Anaesthesiology Research Group, Stavanger University Hospital, Stavanger, Norway; 4 Centre for Clinical Research, Haukeland University Hospital, Bergen, Norway; 5 Department of Clinical Medicine, University of Bergen, Bergen, Norway

## Abstract

**Background:**

Gender bias may represent a threat to resident assessment during surgical training, and there have been concerns that women might be disadvantaged. There is a lack of studies investigating gender differences in ‘entry-level’ real-life procedures, such as laparoscopic appendectomy. We aimed to explore potential gender disparities in self-evaluation and faculty evaluation of a basic surgical procedure performed by junior surgical residents in general surgery.

**Methods:**

A structured training program in laparoscopic appendectomy was implemented before undertaking evaluation of real-life consecutive laparoscopic appendectomies by junior residents in general surgery. Resident and faculty gender-pairs were assessed. Intraclass correlation coefficient (ICC) was calculated using a single-rater, consistency, 2-way mixed-effects model.

**Results:**

A total of 165 paired sessions were completed to evaluate resident–faculty scores for the procedure. Overall, 19 residents participated (43% women) and 26 faculty (42% women) were involved. The overall correlation between faculty and residents was good (ICC > 0.8). The female–female pairs scored higher for most steps, achieving excellent (ICC ≥ 0.9) for several steps and for overall performance. Female residents were more likely to give a higher self-evaluated score on own performance particularly if evaluated by a female faculty. Also, female trainees had highest correlation-score with male faculty.

**Conclusions:**

This study found higher performance scores in female surgical residents evaluated during real-time laparoscopic appendectomy. No negative gender bias toward women was demonstrated. Better insight into the dynamics of gender-based interaction and dynamics in both training, feedback and influence on evaluation during training is needed when evaluating surgical training programs.

## Introduction

Gender disparities and implicit gender bias represent a potential threat to the integrity of resident assessment in medicine [1] and particularly during surgical training [2–6]. Concerns have addressed gender-based bias in granting surgical residents autonomy during training and suggested gender stereotypes to disadvantage women in traditionally male-dominated specialties [4, 7–9].

Female surgical residents are proposed to be at higher risk for the ‘impostor syndrome’ or express lower confidence in their own knowledge and operating skills [10, 11]. However, studies investigating gender differences specifically for ‘entry-level’ procedures during general surgery are lacking. Further, social structure and gender attitudes may differ between geographical regions, with a longer history of gender equity and social acceptance of female role models in some countries. Women have had a comparatively strong position in the Nordic countries over long periods in history. Dating over 1000 years back, Norse mythology and the Viking sagas tell us about the shield-maidens—the Viking women warriors who fiercely fought side-by-side their men—fearless and no less courageous of the battle at hand [12]. Notably, in more modern times, women in Norway and the Nordic countries have been taking up several leadership positions in society. Indeed, Norway is ranked second on the list of countries who have managed to incorporate gender parity, according to the Gender Gap Report of 2020 [13] by the Word Economic Forum. However, how this may contribute to gender parity or not in surgical training is less well investigated.

Thus, we aimed to explore how gender would be related to variation in evaluation of a basic surgical procedure based on a program structured for junior surgical residents. We hypothesized that paired gender setups between resident and faculty would influence the scoring with a likely bias toward women.

## Methods

A structured training program in laparoscopic appendectomy in general surgery rotation was implemented to enhance learning and feedback for junior surgical residents and embedded in real-life surgery over a 12-month period. The detailed curriculum, training with dry-lab simulation and instructor education details are reported in extensive detail elsewhere (Skjold-Ødegaard et al. in revision). Briefly, a standardized approach with focus on educational principles is utilized [14]. Residents in general surgery are supervised by either chief residents or consultant surgeons and each step of the procedure (Table 1) is evaluated on a 6-point score (Table 2), based on the global assessment score (GAS) defined by the extent to which the trainees were dependent on support (e.g., 1 = unable to perform, 5 = unaided (benchmark), 6 = proficient) [15]. All residents and faculty completed the training and (for trainers) the train-the-trainer program.

**Table 1:** The stepwise and standardized approach to the procedure for evaluation

Steps	Evaluation
#1 Abdominal entry	Access through umbilicus, safe incision, trocar insertion (12 mm). Establish pneumoperitoneum
#2 Placement of trocars	Safe placement of trocar in left iliac fossa (12 mm) and in midline above the symphysis pubis (5 mm)
#3 Appendix identification	Inspect all 4 quadrants; identify appendix using atraumatic graspers
#4 Management of the small bowel	Safe handling of small bowel in an atraumatic manner
# 5 Division of the mesoappendix	Use of bipolar diathermy and cold scissors to ensure hemostasis and safe division of mesoappendix
#6 Dividing appendix	Safe placement of ties using two endoloops before transection with cold scissors
#7 Extracting specimen	Safe specimen retrieval using a bag (endobag) via the umbilical trocar. Visual control of appendiceal stump for leak or bleed
#8 Closure	Extracting all ports, safe closure of fascia and skin
#9 Overall assessment	A general score for the whole procedure as evaluated

**Table 2:** Scores rated for each step

Score	Definition
1	Not performed by resident, step had to be done by faculty
2	Partly performed by resident, step had to be partly done by faculty
3	Performed by resident with substantial verbal support from faculty
4	Performed by resident with minor verbal support from faculty
5	Competent performance, safe (without guidance)
6	Proficient performance, ‘could not be better’

The gender-pairings of resident–faculty sessions were assessed for gender influence on the evaluation score of technical steps and for an overall assessment. Each procedure was scored by both the resident and the supervising faculty for each step and for the final overall assessment of the procedure.

Data were collected prospectively from a consecutive cohort of patients undergoing laparoscopic appendectomy over a 12-month period. Only procedures done by junior residents (< 4 years of experience) were included for evaluation. Matched resident–faculty pairings for each gender combination were investigated for the surgical steps (steps 1 to 8 of the procedure) as well as overall performance after the appendectomies.

### Statistics

Computation and graphics were done by R 3.6 (www.r-project.org) and Matlab 9 (Mathworks, Natick, MA). Statistical analyses were run by Social Package for Social Sciences for Mac v. 26 (IBM SPSS; Armonk, NY, USA). Intraclass correlation coefficient (ICC) was calculated using a single-rater, consistency, 2-way mixed-effects model. Nonparametric Spearman’s rho was used to assess correlation in scores between genders. Descriptive data were analyzed using nonparametric tests, with Kruskal–Wallis for analyses of continuous data, or Chi-square for categorial variables. Spearman’s *rho* correlation was reported for nonparametric variables.

A bubble-chart of score-agreement was created to depict correlation between scores for each step and for the overall assessment, whereby the resident score is on the y-axis and faculty score on x-axis. For each square evaluated, the percentage of a green bubble represents the overall agreement between faculty and resident. Bubbles on the line trajectory represents equal scores given by resident and faculty; bubbles on either side of the line represent a higher assessment score given by the resident (if to the left of the line) or the faculty (if to the right of the line). The percentage in the upper left corner represents the rate of residents giving themselves higher scores (than faculty assessed score), the percentage in lower right part of the quadrant is when faculty gives a higher score than the resident. All statistical tests were two-tailed and statistical significance attributed to *P* < 0.050.

## Results

**Fig. 1:**
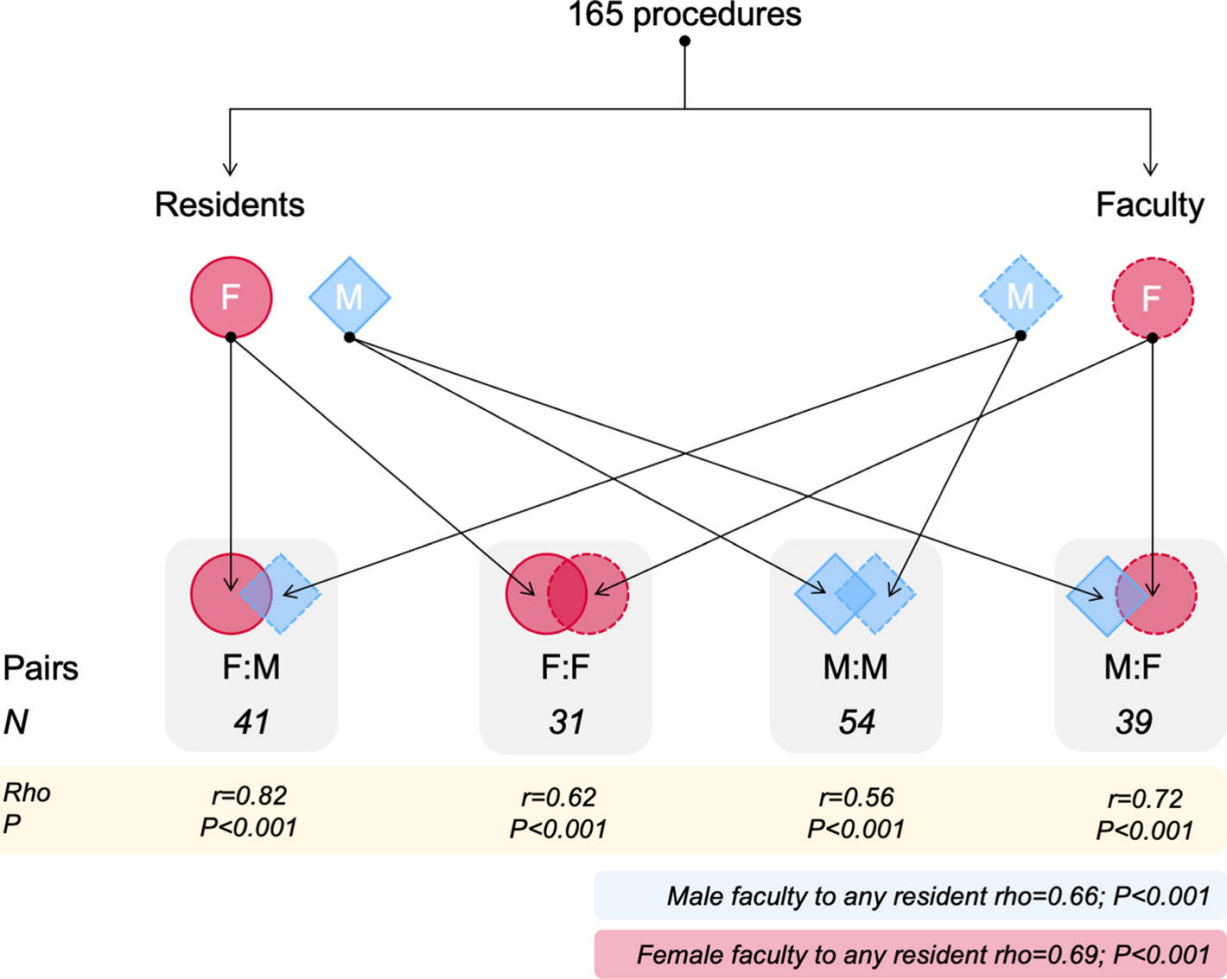
Correlation between gender-pairs of residents and faculty for overall procedure score. Presented is the correlation (rho, r) for overall assessment between gender-pairs. In addition is the correlation given for male faculty to all residents (both genders) and female faculty (bottom right)

**Fig. 2:**
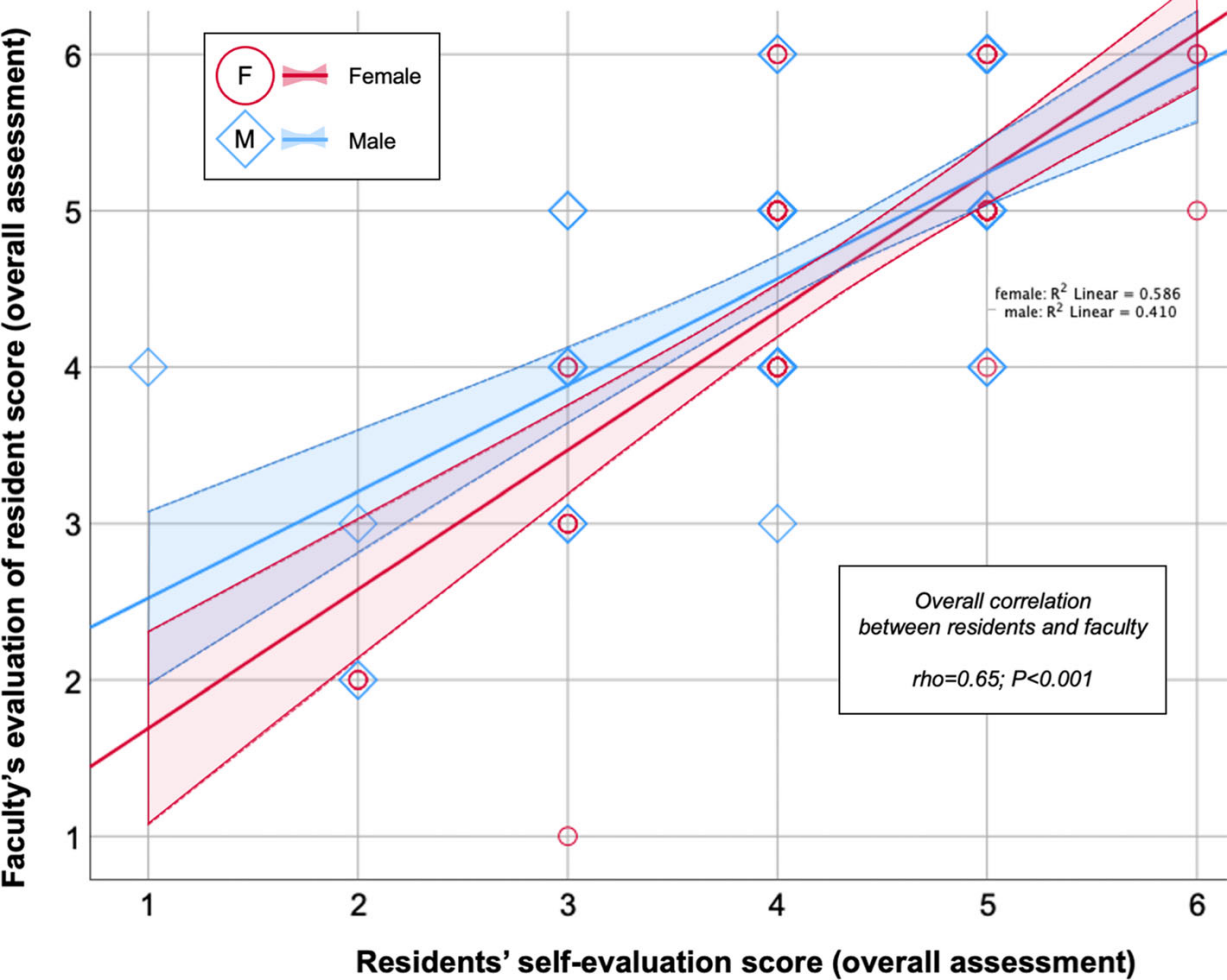
Correlation of overall procedure score between faculty and residents, for men and women**.** Correlation between resident and faculty evaluation of the overall assessment of the procedure and for gender. Shapes of circles (women) and diamonds (men) overlap and each dot may thus represent several scores

During the training program, 165 paired sessions were completed to evaluate resident–faculty scores for the procedure (Fig. 1). Overall, 19 residents participated (of which 43% women) and 26 faculty (42% women) were involved. In most training situations (*n* = 133, 80%), the faculty was a senior resident. The gender distribution of male and female pairs being either a resident or faculty (e.g., F:M, F:F, M:M and M:F combinations; Fig. 1) was not significantly different (Table 3). Distributions of intraoperative findings, number of procedures and operating time were similar between genders, but use of simulator prior to surgery was almost twice as frequent in male residents (Table 3). The overall assessment score between male and female genders demonstrated good correlation (Fig. 2).

**Table 3:** Distribution characteristics according to surgical resident gender

Characteristics	Resident gender		*P*
	Female *n* (%)	Male *n* (%)	
*Faculty gender *(*n*)			0.885
Female *n* (%)	31 (43%)	39 (42%)	
Male *n* (%)	41 (57%)	54 (58%)	
*Intraoperative appendix category*			
Non-inflamed, unclear	7 (10%)	4 (4%)	0.362
Inflamed	55 (79%)	76 (83%)	
Perforated	8 (11%)	12 (13%)	
*Procedure volume *			
Median (IQR)	21 (10-35)	18 (7-34)	0.231
≤ 30 laparoscopic appendectomies	52 (72%)	66 (71%)	0.859
> 30 laparoscopic appendectomies	20 (28%)	27 (29%)	
*Operating time* (minutes)			
Median (IQR)	60 (55-70)	60 (45-75)	0.140
Used simulator *			
No	50 (83%)	44 (67%)	0.032
Yes	10 (17%)	22 (33%)	
Used web tools *			
No	37 (63%)	41 (59%)	0.704
Yes	22 (37%)	28 (41%)	

Data are reported as medians and interquartile ranges (IQR) or number with rates (%)

Numbers/percentages may not add up due to missing data or rounding

^*^ Within the last week prior to surgery; missing data in 39 cases (sim) and 37 cases (web)

**Fig. 3:**
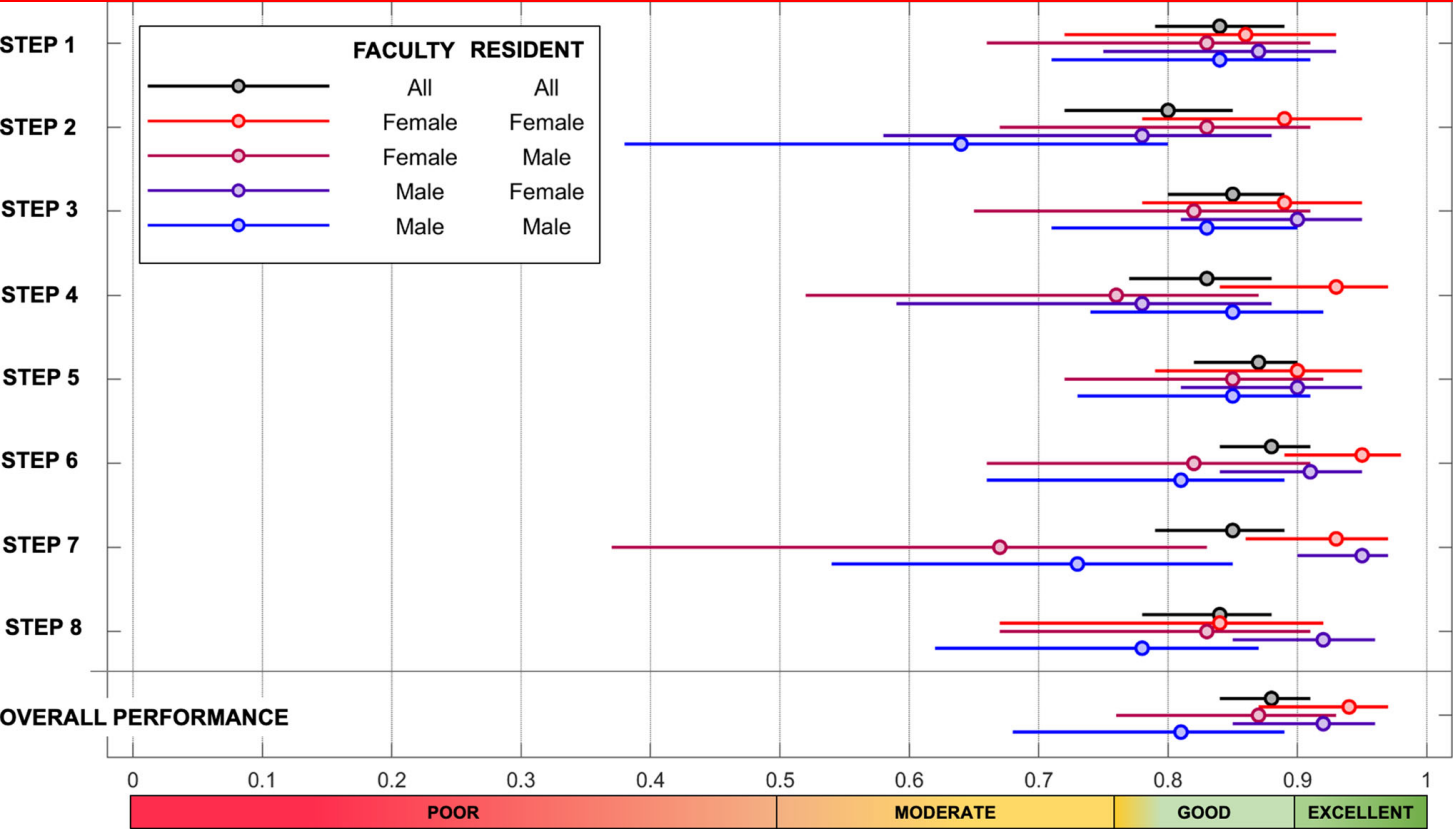
Correlation between faculty and residency gender-pairs for each step. See Table 1 for explanation to each step. An overall impression score of the procedure was given (bottom part). Intraclass correlation coefficient (ICC) between gender groups with 95% confidence intervals

The intra-correlation class (ICC) for faculty–resident gender-pairs for each procedure step is presented in Fig. 3. The overall correlation between faculty and residents was good for most steps, while bordering toward moderate for placement of ports (step 2). Female–female pairings had higher correlation scores for port placement, identification of the appendix, transection of the appendix and extraction of specimen (steps, 2, 4, 6 and 7, respectively). The female–female pairs scored higher overall for most steps and had a higher number of steps with excellent correlation, compared to male–male pairs (Fig. 3). Also, male–male pairs had only moderate-to-good ICC for 3 steps, while no such low scores were noted for female–female pairs. Male–male pairs had the lowest overall ICC, while female–female had the highest ICC (Figs. 1 and 3).

**Fig. 4:**
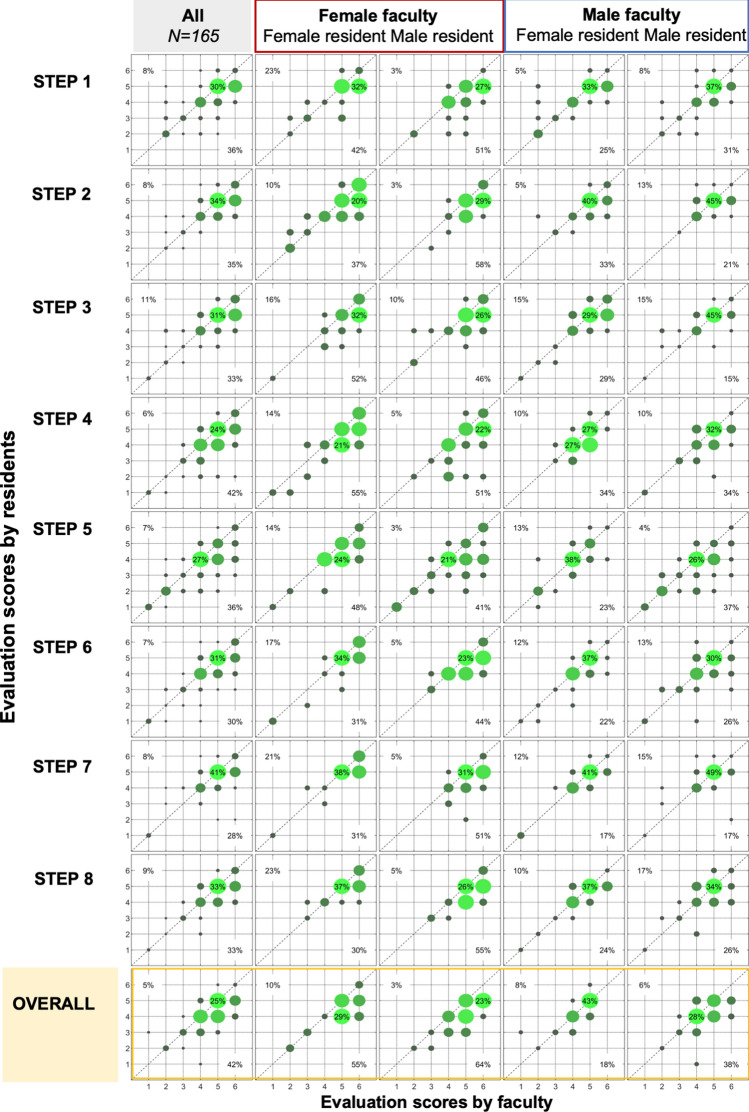
Self-assessment by residents according to gender and to the faculty assessment. For each square, the upper left part of the diagram is when the resident self-rated score is higher compared to that of faculty for skills evaluation; on the lower right part in each square is where the faculty rated score is higher than that of the resident. Bubble size and percent indicate agreement between resident and faculty in score

In 70 (42%) of the sessions, the faculty was female and of these paired with female resident in 31(44%; Fig. 4). Male faculty supervised 95 sessions with female residents in 41 (43%). Overall, the faculty scored the residents consistently higher compared to residents’ own scores (Fig. 4). Female faculty scored residents’ skills higher compared to the residents’ self-evaluation score. Notably, the group most likely to give a higher self-evaluated score on own performance was female residents evaluated by a female faculty (Fig. 4).

The widest discrepancy in scores was found for male residents (evaluated by both female and male faculty) for placement of ports; extraction of specimen; closure of fascia and skin (steps for 2, 7 and 8).

## Discussion

In the current study, we found an overall good (bordering on excellent for ICC) agreement in evaluation of procedure skills between faculty and residents for an entry-level procedure such as laparoscopic appendectomy. However, female–female pairs more often had excellent agreement in skills evaluation, with male–male pairs more often agreeing only moderately. Female surgical residents were more likely to give high self-rated scores that correlated with the rating of the female faculty, but female residents also scored higher with male faculty. The lowest performance scores were noted for male–male pairs.

The current findings are in contrast to the gender bias reported from other regions and surgical specialties, typically reporting lower scores and negative male–women associated evaluations [2, 4, 16–18]. One study found that, even both men and women performed equally across several items, general surgery residents generally tended to underscore their own evaluation (when compared to faculty) and women did this to a greater extent [19]. A more recent study among urologist trainees also found that trainees tended to underrate performance and technical skills, but this was more common among women, whereas men tended to score themselves similar to the expert raters [20]. A similar pattern was found among plastic surgery trainees [21]. Hence, previous studies have demonstrated an overall lower score rating among women across technical and non-technical domains in general surgery, in urology and plastic surgery. This gender difference is in contrast to the current study, in which we demonstrate an overall good agreement between genders and trainee–trainer pairs, but slightly better (but not overrated) for women residents and faculty. In the current study, the female residents seemed also to confidently self-evaluate themselves higher compared to male residents, but in accordance with the faculty evaluation. There are no clear explanations to this finding. Of note, male residents reported twice as often to have used the simulation tools available in the department for ‘dry training’ prior to laparoscopic appendectomy compared to the female residents. We have no clear explanations to this gender discrepancy at the current time, yet believe the overall use of simulation was low at 25% of all procedures. Barriers to this need to be investigated (lack of time or availability; no incentives prior to real surgery; perceived self-sufficient in procedure or the like). Also, we did not collect information on adjunct procedures done and overall experience or volumes for each resident. This would be of interest in the future to possibly view the added spill-over effect from related training experience.

Previous studies have for the most part been conducted in a North American training system, in which most findings point to a systemic bias against female residents in training [7, 19–21]. While a perceived bias of skills may be present in medical students, a systematic review found no difference in skills acquisition between gender in surgical residents [22]. Small but significant gender differences in motivation and personality have been demonstrated in surgical residents [23], but overall surgical residents are a comparable and relatively homogenous group in their motivation and drive toward success in the profession. Other factors may be more important in self-evaluation of skills. A small, pilot study suggested that emotional intelligence was associated with better accuracy for self-assessment of surgical quality and expert score given in a simulation study on laparoscopic appendectomy [24]. Another small study also found self-assessment to be more important for non-technical skills and formative development among residents [25]. Nonetheless, the good correlation in the current study of laparoscopic appendectomy may suggest that the validated score as previously reported and validated [15] is valid also for a procedure done early in surgical training.

Based on the current study, we speculate if the existing Scandinavian social structure may have an influence on gender perception even in surgical training. Obviously, this needs wider scale validation to be confirmed. However, all Nordic countries are in the top list of countries scoring high on gender parity evaluation by the World Economic Forum [13]. While the gender parity is overall favorable in the Nordic region, the recruitment of women to general surgery has been slow up until recent times [26, 27] with general surgery being seen less attractive as a career option among female medical students [28]. Hence, the influence of social structure promoting gender equity may only partially explain the results in the current study. Also, we appreciate that the results apply to one department and one set of junior trainees only. Thus, further investigation is needed to explore similar effects in advanced surgical training or even into consultancy.

The study results stand in contrast to studies demonstrating lower confidence and a trend toward self-underestimating performance among female residents [20, 21, 29]. The current study suggests women to have a high self-rated score but with good correlation of faculty scores, hence it does not seem to be the result of an inflated self-perceived ability but rather represents a true match in scoring of performance. The results may also highlight the positive effect of female–female mentorship roles and, possibly, may point to a need for focused educational training in male–male paired scenarios [30]. However, while the male–male scores may stand out as the lowest scores, several factors may explain this, and the current study was not constructed to identify causality in gender variation.

No previous studies on surgical training by gender stem from the Nordic countries in this regard as we know of. Better insight into the dynamics of gender-based interaction and dynamics in both training, trainer feedback and trainee influence on evaluation during training is needed when evaluating surgical training programs. Also, the perceived poor correlation with male–male paired training and supervision may suggest that particular focus may be warranted to better understand how male–male pairs perceive education and training in basic surgical skills. Furthermore, granular discrepancies in some of the steps of laparoscopic appendectomy may give room for improved understanding of technical steps that warrant further simulation and focused training to achieve proficiency level among trainees.

Some limitations should be noted. One is that we have no formal evaluation of the educational skills of the faculty other than completion of the train-the-trainer course, nor any baseline description of the junior residents evaluated in the current study. However, all faculty went through a train-the-trainer program with focus on educational didactics and several consultant surgeons have also completed the LapCo training[31]. Also, all residents went through a theory program with a subsequent dry-lab simulation before entering the real-life surgery. We do not know how prior experience may have influenced the scores or may have been attributed to gender. Further, a laparoscopic appendectomy may be easy or difficult, depending on both patient attributes (such as body weight and composition) as well as disease characteristics (e.g., mildly or grossly inflamed; retrocecal position of the appendicitis). Perforated appendicitis rates were similar between residents. Also, only laparoscopic appendectomies deemed eligible for residents to perform were evaluated, hence reducing the potential risk of a difficulty bias between genders. Further, blinded evaluation could be formed for the videorecorded part of the procedure as this has been demonstrated as an effective learning tool [32], but this would exclude the interaction during the procedure and non-video recorded details, such as abdominal entry, port placements, and fascia and skin closures.

The generalizability of the findings remains to be demonstrated in a wider context. However, being one of the largest units in general surgery in the country with a high volume of general surgery procedures and number of trainees, we believe the findings to be of wider interest and applicability. It also serves as a balanced report to the ongoing gender equity and disparity debates [3, 7, 11, 33–35]. In the current study, we report how female residents in general surgery perceive their performance for an entry-level procedure such as laparoscopic appendectomy. The study substantiates the statement by others, that ‘quality surgical training of women and men is far more similar than different, and individual personalities and learning styles supersede generalizations’ [36]. Indeed, understanding differences in personality may be more important than difference in sex. We support the sentiment that gender diversity and gender equity should be promoted across all levels of surgery. However, specific attributes to teaching and education should go beyond true and perceived gender biases to understand how to best teach and train skills to enhance performance in general surgery residents [37].
